# Dr. Hayward on the Statistics of Pulmonary Consumption

**Published:** 1843-02-18

**Authors:** 


### DR. HAYWARD ON THE STATISTICS OF PULMONARY CONSUMPTION.

It is well ascertained that this formidable disease was on the increase in Great Britain from the beginning to the middle of the last century, and that it remained stationary from that time to the year 1837, the period when the calculation was made. This increase was greatest among the middling and upper classes of society; in fact, the lower classes, it was thought, did not suffer more from it than formerly; which was perhaps to be attributed to an improvement in their mode of living. This increase is the more remarkable, as the mortality from other diseases has lessened in a striking degree in the same period of time. In the year 1700, the number of yearly deaths in every 1000 inhabitants in the city of London, from all diseases, was 31, and only 4 of these were from consumption; while in 1821, the number from all diseases was only 19 in 1000, and 6 1-5 of these were from consumption. From that period to the year 1837, the proportion of deaths from these two sources remained the same.

To ascertain if a similar condition of things obtained in this country, Dr. H. has examined the bills of mortality of Boston, New York and Philadelphia from 1811 to 1840, a period of thirty years.

The most striking fact brought to light by these tables, is the great decrease of deaths by consumption in these cities. This descrease has been great in all, but greater in Boston than in either of the others; and this is not only a relative, but an absolute decrease, for the mortality has been somewhat more during the last ten years than it was thirty years ago. At that time the deaths were about 22 to every 1000 inhabitants; and now they are nearly, if not quite, 23 to 1000.

It appears, that during the whole period embraced in these tables, Philadelphia has suffered less from consumption than either of the other cities; the average number of deaths from that disease for the whole time being as 1 in 7.003 of the whole number; while in Boston they were as 1 in 6.185; and in New York as 1 in 5.547. But during the last ten years, Boston has enjoyed the greatest exemption. From 1831 to 1840 inclusive, the deaths in Boston from consumption were only 1 in 7.587, in Philadelphia 1 in 7.482, and in New York 1 in 5.952.

It will be seen, by examining the bills of mortality of the city of Boston, that there has been a very striking and uniform improvement as to pulmonary consumption since the year 1811. By the United States census of 1810, Boston contained 33,250 inhabitants; in 1820, 43,298; in 1830, 61,392; and in 1840, 93,452. In 1811, when the population had not probably increased at all from the preceding year, as it was a period of great depression in commercial affairs, the whole number of deaths was 742, of which 221 were of consumption; while in 1840, with a population nearly three times as great, and with nearly three times as many deaths, there were only 19 more fatal cases of consumption, the whole number being but 240; not quite one in 8 of all the deaths, and not 3 in 1000 inhabitants.

Dr. Hayward contends that this decrease of mortality in consumption in Boston is real, and is not attributable to improved diagnosis as some have contended. He thinks it should be referred to a combination of causes, rather than to any single one.

These I should say were mainly the great improvements that have taken place in living during the last thirty years; to the increased comforts of life, which are now enjoyed by every class of the community. People are better fed, better clothed, live in more comfortable houses, indulge less in excesses of all kinds, and pay more attention to personal cleanliness, than they formerly did. They adopt better and more effectual means to protect themselves from the vicissitudes of temperature, and the low rates at which cotton fabrics can be obtained, and the consequent general use of them, have no doubt contributed essentiallly to this desirable result.

*N. E. Quar. Journ. Med. and Surg.* Jan. 1843.

